# lncRNA *EGOT* Is the Marker of HPV Infection and a Prognostic Factor for HNSCC Patients

**DOI:** 10.3390/biomedicines13040798

**Published:** 2025-03-26

**Authors:** Tomasz Kolenda, Piotr Białas, Kacper Guglas, Maciej Stasiak, Joanna Kozłowska-Masłoń, Karina Tylkowska, Anna Zapłata, Paulina Poter, Marlena Janiczek-Polewska, Patrycja Mantaj, Paulina Gieremek, Urszula Kazimierczak, Anna Przybyła, Katarzyna Regulska, Beata Stanisz, Ewa Leporowska, Andrzej Mackiewicz, Jacek Mackiewicz, Joanna Kazmierska, Zefiryn Cybulski, Anna Teresiak

**Affiliations:** 1 Research and Implementation Unit, Greater Poland Cancer Centre, Garbary 15, 61-866 Poznan, Poland; kacper.guglas@wco.pl (K.G.); katarzyna.regulska@wco.pl (K.R.); zefiryn.cybulski@wco.pl (Z.C.); anna.teresiak@wco.pl (A.T.); 2 Microbiology Laboratory, Greater Poland Cancer Centre, Garbary Street 15, 61-866 Poznan, Poland; kertylkowska1207@gmail.com; 3 Department of Cancer Immunology, Chair of Medical Biotechnology, Poznan University of Medical Sciences, 8 Rokietnicka Street, 60-806 Poznan, Poland; maciej.stasiak96@gmail.com (M.S.); ukazimierczak@gmail.com (U.K.); andrzej.mackiewicz@wco.pl (A.M.); 4 Department of Diagnostics and Cancer Immunology, Greater Poland Cancer Centre, 15 Garbary Street, 61-866 Poznan, Poland; 5 Department of Cell Biology, Poznan University of Medical Sciences, 5D Rokietnicka, 60-806 Poznan, Poland; 6 Laboratory of Cancer Genetics, Greater Poland Cancer Centre, 15 Garbary Street, 61-866 Poznan, Poland; joanna.kozlowska@wco.pl; 7 Faculty of Biology, Institute of Human Biology and Evolution, Adam Mickiewicz University, Uniwersytetu Poznańskiego 6, 61-614 Poznan, Poland; 8 Faculty of Biology, Adam Mickiewicz University, Umultowska 89, 61-614 Poznan, Poland; 9 Department of Laboratory Diagnostics, Greater Poland Cancer Centre, 15 Garbary Street, 61-866 Poznan, Poland; anna.zaplata@wco.pl (A.Z.); ewa.leporowska@wco.pl (E.L.); 10 Department of Oncologic Pathology and Prophylaxis, Poznan University of Medical Sciences, Greater Poland Cancer Center, 15 Garbary Street, 61-866 Poznan, Poland; paulina.poter@gmail.com; 11 Department of Clinical Oncology, Greater Poland Cancer Centre, 15 Garbary Street, 61-866 Poznan, Poland; marlena.janiczek@wco.pl; 12 Department of Electroradiology, Poznan University of Medical Sciences, 61-701 Poznan, Poland; 13 Radiation Protection Department, Greater Poland Cancer Centre, 15 Garbary Street, 61-866 Poznan, Poland; patrycja.mantaj@wco.pl; 14 Departament of Pharmaceutical Chemistry, Poznan University of Medical Sciences, Rokietnicka Street 3, 60-806 Poznan, Poland; paulina.gieremek@wco.pl (P.G.); bstanisz@ump.edu.pl (B.S.); 15 Pharmacy, Greater Poland Cancer Centre, Garbary 15, 61-866 Poznan, Poland; 16 Department of Medical and Experimental Oncology, Institute of Oncology, Poznan University of Medical Sciences, 60-512 Poznan, Poland; jmackiewicz@ump.edu.pl; 17 Radiotherapy Department II, Greater Poland Cancer Centre, 15 Garbary Street, 61-866 Poznan, Poland; joanna.kazmierska@wco.pl

**Keywords:** *EGOT*, HNSCC, lncRNAs, HPV

## Abstract

**Background:** High-risk human papillomavirus (HPV) contributes to oropharyngeal cancers through mechanisms involving the deregulation of host cell functions by oncoproteins E6 and E7. Changes in the epigenome, particularly involving long non-coding RNAs (lncRNAs), are crucial for understanding HPV-related carcinogenesis. **Methods:** This study aimed to analyze the expression levels of lncRNAs in HPV-related head and neck squamous cell carcinoma (HNSCC) to determine their biological and clinical significance, addressing the current gap in clinically validated biomarkers for early screening and therapeutic interventions. **Results:** The study highlights the significant overexpression of the *EGOT* gene in HPV-positive HNSCC samples, suggesting its potential as a marker to distinguish between HPV-negative and HPV-positive cases. Furthermore, high *EGOT* expression correlates with better overall survival (OS) and indicates possible resistance to therapy, making it a valuable prognostic factor. **Conclusions:** These findings underscore the potential of incorporating *EGOT* expression analysis in clinical practice for improved patient stratification and treatment outcomes in HNSCC.

## 1. Introduction

Head and neck squamous cell carcinomas (HNSCC) represent a serious global health issue. The incidence and mortality rates of HNSCC vary significantly by geographic region and demographic characteristics [1,2,3]. The International Agency for Research on Cancer (IARC) of the World Health Organization has indicated a diverse range of risk factors associated with HNSCC. High tobacco and alcohol consumption rates, and exposure to environmental pollutants, such as asbestos, formaldehyde, and other industrial chemicals, have been linked to an increased risk of HNSCC [4,5]. Moreover, infections with viral agents, particularly human papillomavirus (HPV) and Epstein-Barr virus (EBV), are strongly related to certain types of HNSCC [6,7,8]. Clinical data point out that HPV is notably linked to oropharyngeal cancers, while EBV is associated with nasopharyngeal carcinomas [9]. Furthermore, the demographic characteristics influencing HNSCC include age, gender, and socioeconomic status [10]. HNSCC is more common in men, and the risk of incidence increases with age [11]. It should be noted that, despite improvements in treatment schemes and diagnostic methods, HNSCC remains one of the most deadly types of cancer worldwide, including in Poland [12,13,14,15,16,17]. HPV-positive (HPV(+)) HNSCC is associated with number of oral sex partners, HPV infections, younger age, oropharynx location (base of tongue, tonsil), increased survival, and sensitivity to radiochemotherapy. On the other hand, HPV- negative (HPV(−)) HNSCC is associated with use of alcohol and tobacco, older age, and worse survival [18].

Within the Papillomaviridae family, roughly 200 HPV genotypes have been identified based on viral genome sequences and classified as high-risk and low-risk HPVs. The high-risk HPV group includes types 16, 18, 31, 33, 35, 39, 45, 51, 52, 56, 58, and 59 and low-risk HPV group includes types 6, 11, 40, 42, 43, 44, 54, 61, and 70 [19,20,21]. After exposure to HPV, the virus enters and is uncoated in the host cell. The circular viral DNA is then translocated to the nucleus, where it uses host cell enzymes to replicate its genome along with host chromosomes. This spectrum of cellular processes deregulates the normal biological functions of host cells [22]. It has been documented that constant low-copy replication of the viral genome in basal keratinocytes is one of the mechanisms by which HPV evades the host immune system [22]. In the case of high-risk (oncogenic) HPV, cell cycle dysregulation by the oncoproteins E6 and E7 constitutes the initial step driving HPV-related carcinogenesis [23]. While the role of protein-coding genes is well known, changes in the epigenome, such as those involving the functioning of long non-coding RNAs (lncRNAs), are not precisely defined. In recent years, studies have indicated that lncRNAs could be a crucial factor in the regulation of tumor microenvironment and may be used for the improvement of HNSCC immunotherapies. Several lncRNAs were described as immune-related, such as *AL139158.2*, *AL031985.3*, *AC104794.2*, *AC099343.3*, *AL357519.1*, *SBDSP1*, *AS1AC108010.1*, and *TM4SF19-AS,* the expression of which is associated with longer overall patient survival. On the other hand, a worse prognosis was correlated with the expression of *MiR31HG*, *TM4SF19-AS1*, and *LINC01123*. It should be noted that cancer cells and immune cells as well as cancer-associated fibroblast can interact using lncRNA. For instance, lncRNA *HOTAIR* is produced by cancer cells and, through exosomes, translocates into macrophages and causes their polarization into M2 type. It was indicated that lncRNA *CRNDE* is associated with pro-tumor microenvironment and its role is connected with depletion of CD8+ T-cells and suppression of their cytotoxicity [24]. *MALAT-1*, *HOTAIR*, and *XIST* have been identified as lncRNAs with significant diagnostic and prognostic potential in OSCC. *MALAT-1* plays a crucial role in modulating key signaling pathways, including *Wnt/β-catenin*, *NF-κB*, and *PI3K/AKT/mTOR*. *HOTAIR* exerts its regulatory effects through *EZH2*, while *XIST* influences OSCC progression by modulating *miR-29* and affecting the *p53*-mediated apoptosis pathway [25]. Masrour et al., based on the available literature, included only 25 studies in their meta-analysis. However, they did not reach any definitive conclusions regarding the selection of specific lncRNAs that could form a reliable diagnostic panel [26]. This highlights the need for further research on lncRNAs and underscores that their clinical implementation remains an open question. Similarly, Guo et al. reviewed the literature on non-coding RNAs in HPV(+) HNSCC and summarized five studies with inconsistent findings regarding lncRNAs. Notably, some of the HPV-related lncRNAs discussed in their analysis were originally identified in cervical cancer models [27].

Therefore, there is an unmet need to understand changes in the regulatory mechanisms of the transcriptome. This understanding will likely contribute to a better knowledge of the biology of HPV-positive cancers. It may enhance treatment and diagnostic processes, mainly due to the lack of clinically validated biomarkers for early screening [28,29].

This study aimed to analyze lncRNAs using patient’s samples and qRT-PCR profiling method to quantify expression levels of ninety lncRNAs connected with cancerogenesis and find the differences between HPV(+) and HPV(−) patients. Based on this, and using the TCGA data collected for HNSCC patients, we analyzed the HPV(+) individuals to determine the biological and clinical significance of HPV viral-related lncRNAs.

## 2. Methods

### 2.1. PART I—In Vitro Analyses

#### 2.1.1. Patient Cohort in PART I

In this study, 14 patients with a median age of 63 years old were enrolled, and we obtained Formalin-Fixed Paraffin-Embedded Tissue (FFPET) samples of patients who underwent surgical procedures at Heliodor Swiecicki Clinical Hospital in Poznan between 2016–2018. FFPET samples were used in the routine diagnostic procedures. Complete pathological reports were available, detailing the TNM classification and tumor grade. The HPV infection status of the tissue samples was determined using p16INK4A (p16) immunohistochemistry (IHC) CINtec^®^ p16 Histology (Roche Diagnostics; Indianapolis, IN, USA) antibody according to manufacturer’s protocol, with twelve samples testing negative and two samples testing positive for HPV (Table 1).

#### 2.1.2. RNA Isolation and Reverse Transcription Reaction

According to the isolation protocol, total RNA was isolated using a High Pure FFPET RNA Isolation Kit (Roche). The concentration and purity of samples were determined spectrophotometrically (NanoDrop 2000, Thermos Fisher, Waltham, MA, USA) by measuring A = 260 nm, A = 280 nm, and A = 230 nm, and the ratios A260/280 nm and A260/230 nm were calculated. A total of 1.0 μg RNA was used to prepare cDNA in reverse transcription reaction according to producer protocol LncProfiler qPCR Array Kit (SBI).

#### 2.1.3. Quantitative Real-Time PCR

Quantitative real-time PCR reactions were performed using the LightCycler 480 system (Roche, Atlanta, GA, USA). To analyze the expression levels of selected lncRNAs, the LncRNA Profiler qPCR Array Kit (Human) (System Bioscience SBI, Tokyo, Japan), and LightCycler 480 SYBR Green I Master (Roche) were used. The housekeeping genes *18S rRNA*, *RNU43*, *GAPDH*, *LAMIN A/C*, and *U6* served as internal controls. Gene expression was normalized against the mean value of used housekeeping genes to determine the relative expression levels of the genes of interest (Supplementary Table S2).

#### 2.1.4. Statistical Analyses in PART I

All statistical analyses were conducted using Prism 5.0 (GraphPad Software) and Statistica 12.0 (StatSoft). The Shapiro–Wilk test was employed to assess data normality. The Student’s *t*-test was used to calculate the level of statistical significance (*p*-value). A *p*-value of less than 0.05 (*p* < 0.05) was considered statistically significant for comparing cancer samples and healthy subjects, as described previously [30].

### 2.2. PART II—In Silico Analyses

Based on the results of in vitro profiling of 90 lncRNAs, 4 potential lncRNAs, *EGO-A*/*EGO-B*, *HAR1B*, *HOXA-AS2,* and *GAS5 family*, were selected, despite the lack of expression in the HPV(+) group. Then, the expression levels of these lncRNAs were checked based on the TCGA database for HNSCC patients. It should be noted that the database contained data for lncRNA *EGOT* (*EGO-A* and *EGO-B* are isoforms of *EGOT*), no data were found for *HOXA-AS2*. Based on the results obtained in the “In Vitro Analyses” section, lncRNA *EGOT* was selected for further analysis presented in the “In Silico Analyses” section.

#### 2.2.1. Patient Cohort

Gene expression data from 567 HNSC patients, sourced from The Cancer Genome Atlas (TCGA) database, was utilized for in silico analysis of the selected lncRNAs (https://xenabrowser.net/datapages/, Xena Browser; accessed on 2 January 2020) [31]. Additionally, clinical data regarding overall survival (OS; *n* = 487) and disease-free survival times (DFS; *n* = 118) were obtained from cBioPortal (https://www.cbioportal.org/; accessed on 2 January 2020). HPV status was available for all patients with OS data, and in the further step, this group was categorized into two subgroups, i.e., HPV-positive (HPV+) and HPV-negative (HPV−).

#### 2.2.2. Statistical Analyses

Similar to in vitro analyses, all statistical analyses were conducted here using Prism 5.0 (GraphPad Software) and Statistica 12.0 (StatSoft). A *p*-value of less than 0.05 (*p* < 0.05) was considered statistically significant in all conducted analyses.

Clinical data and expression levels were obtained from the TCGA databases for *EGOT* (primary transcripts from which *EgoA* and *EgoB* are generated), *HAR1B*, and GAS5. There was no data for *HOXA-AS2* (HOXA3as).

Among subgroups, gene expression analyses of *EGOT*, *HAR1B*, and *GAS5* were conducted using the Student’s *t*-test for unrelated variables. To determine the ability to distinguish HPV-positive (HPV+) from HPV-negative (HPV−) tissue by measuring the expression levels of *EGOT*, *HAR1B*, and *GAS5*, we performed a classification quality assessment using the receiver operating characteristic (ROC) curve. The area under the curve (AUC) was determined to evaluate the performance of this classification.

The analysis of HNSCC patients with HPV-negative (HPV−) and HPV-positive (HPV+) profiles, categorized by high and low *EGOT* expression, was conducted using Fisher’s exact test. Patients were classified based on clinical parameters, including age (under 60 vs. 60 and above), gender, alcohol consumption, cigarette smoking, cancer grade (G1–G4), tumor stage including (T1–T4) and nodal status (N0–N3), perineural invasion status, and lymphovascular invasion presence (presence of tumor cells within definite endothelial-lined spaces, vascular or lymphatic). The smoking group included patients who were actively smoking at the time of data collection and those who had quit smoking less than 15 years prior, corresponding to categories 2 and 4 according to NIH classifications. The non-smoking group included never-smokers and former smokers who had quit smoking more than 15 years prior to data collection, corresponding to categories 1 and 3 according to NIH classifications. Individual parameters were analyzed using the Student’s *t*-test or the Mann–Whitney U test.

Kaplan–Meier estimator was used to plot the survival curves. OS and DFS were assessed using the log-rank test (Mantel–Cox) and the Gehan–Breslow–Wilcoxon test. Pearson and Spearman’s rank correlation coefficient (R) was utilized to evaluate correlations between groups, and a moderate and strong correlation was defined as being above 0.3 or less than −0.3, respectively. Subsequently, pathway analysis was conducted using the Reactome database (reactome.org, accessed on 2 January 2020) to determine the pathways in which the genes participate. The gene cluster analysis in Reactome evaluates whether there is an overrepresentation of specific signaling pathways within a given set of genes. This analysis relies on the hypergeometric distribution of the data.

**Figure 1:**
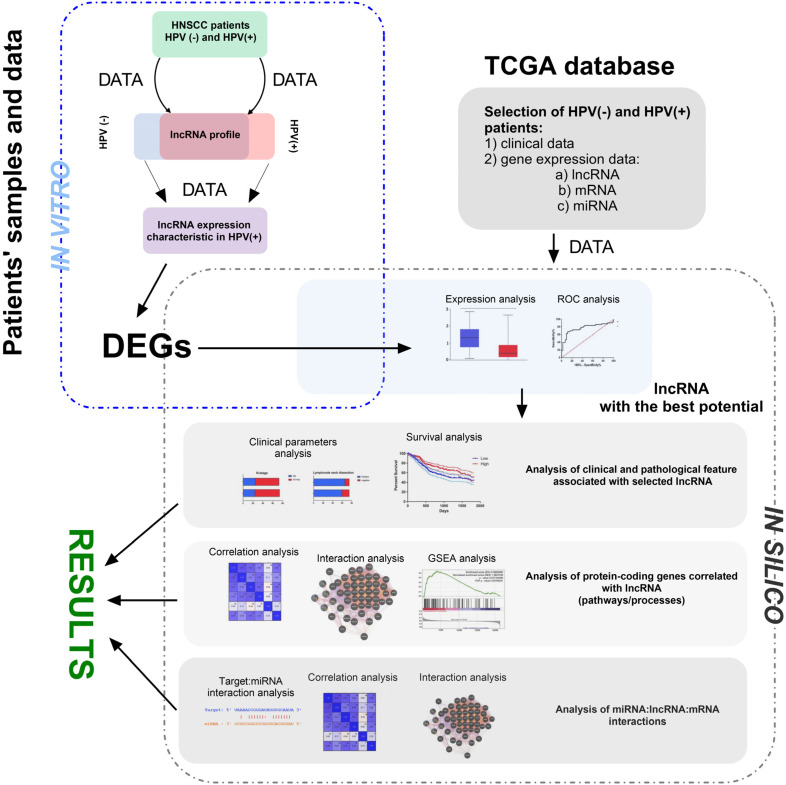
The main steps of the presented study’s methodology, including in vivo and in silico parts. During in vivo analysis, RNA was isolated from FFPET of HNSCC patients and profiled using qRT-PCR method. Next, HPV(+) and HPV(−) groups of patients were compared and DGEs were chosen for the next step. Based on the TCGA data, DGEs were checked and selected lncRNA with the highest discrimination ability between HPV(+) and HPV(−) groups of patients. During in silico analysis, the first step analysis of clinical and pathological features associated with selected lncRNA was performed. Next, analysis of protein-coding genes correlated with lncRNA was performed for description of pathways and processes. Finally, interactions between miRNA:lncRNA:mRNA were analyzed. DEGs—differentially expressed genes; TCGA—The Cancer Genome Atlas; ROC—receiver operating characteristic; GSEA—Gene Set Enrichment Analysis.

Gene Set Enrichment Analysis (GSEA) was conducted using the software available at gsea-msigdb.org; accessed on 2 January 2020. Patients were categorized into groups based on low and high gene expression, as well as HPV-positive and HPV-negative profiles. The expression profiles of all genes for each patient were obtained and analyzed from the University of California, Santa Cruz (UCSC) database. The results were presented as a normalized enrichment score (NES). A pathway or process was considered significantly enriched in a group if it met the following criteria: a nominal *p*-value < 0.05 and a false discovery rate (FDR) *q* < 0.25. All analyses were made similarly as described by us previously [32,33,34]. The main steps of methodology used in our study are summarized in Figure 1.

## 3. Results

### 3.1. Expression of lncRNA EGOT Is Up-Regulated in HPV Positive HNSCC Patients

**Figure 2:**
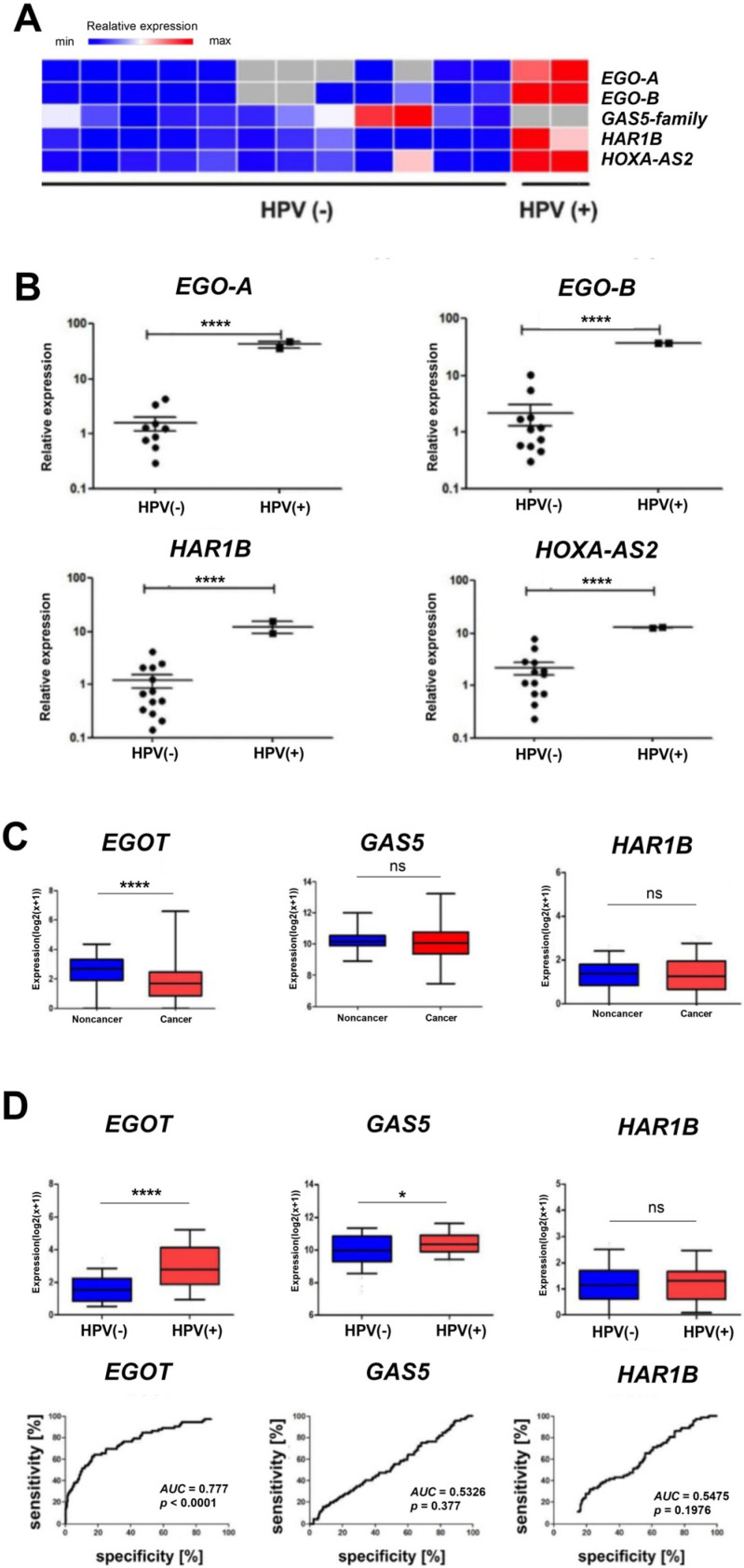
Heat map showing the expression of *EgoA*, *EgoB*, *GAS5*-family, *HAR1B*, and *HOXA-AS2* (*HOXA3as*) in HPV(+)and HPV(−) patient groups (**A**). Expression levels of *Ego*A, *EgoB*, *HAR1B*, and *HOXA-AS2* (*HOXA3as*) in HPV(+) and HPV(−) patient groups (**B**–**D**). A paired *t*-test was used to calculate *p*-values. ROC curve and AUC curve for the expression level of *EGOT*, *GAS5*, and *HAR1B* in HPV(−) and HPV(+) samples (**D**). *p*-values ≤ 0.05 considered significant; ns—not significant; * *p* ≤ 0.05; **** *p* ≤ 0.0001 are considered as significant.

In vitro analyses based on quantitative PCR revealed statistically significantly higher expression levels (*p* < 0.0001) of *EgoA*, *EgoB*, *HAR1B*, and *HOXA-AS2* (*HOXA3as*) among ninety selected long non-coding RNAs in HPV-positive (HPV+) patients compared to HPV-negative (HPV−) patients. In contrast, no expression of the *GAS5* family was observed (Figure 2A,B). For *EGOT*, a statistically significant difference was observed between the expression levels in healthy and cancerous tissue (*p* < 0.0001) based on in silico analyses of a cohort of 487 patients. However, no statistically significant difference between healthy and neoplastic tissues was observed for GAS5 and *HAR1B* (Figure 2C). Additionally, higher expression of *EGOT* was observed in the HPV(+) group of patients compared to the HPV(−) group (*p* < 0.0001, Figure 2D). The ROC curve analysis was used to evaluate the ability to distinguish between HPV(+) and HPV(−) patients based on the expression of *EGOT*, *GAS*5, or *HAR1B*. The area under the curve (AUC) for *EGO*T was 0.777 (*p* < 0.0001), indicating a fair discriminatory ability. In contrast, the AUC for *GAS5* was 0.5326 (*p* = 0.3770), and for *HAR1B,* it was 0.5475 (*p* = 0.1976), indicating a lack of discriminatory power (Figure 2D).

Then, the cohort of 487 patients was stratified into two subgroups based on HPV infection status. Among these subgroups, patients were classified into *EGOT* low and *EGOT* high groups based on median expression level. The difference in the number of patients with HPV(+) and HPV(−) profiles was significantly greater (16/242) in the group with low *EGOT* expression compared to the group with high *EGOT* expression (56/173), with statistical significance (*p* < 0.0001). Consequently, the number of patients with the HPV(+) profile increased in the group with high *EGOT* expression, while the number of HPV(−) patients decreased (Supplementary Figure S1).

In the subsequent analysis, the expression level of *EGOT* was correlated with clinical features. The results demonstrated a statistically significant association between *EGOT* expression and patient age (*p* = 0.011). Additionally, a significant correlation was observed between tumor size and *EGOT* expression within the HPV(+) cohort (*p* = 0.0492). However, no significant differences in *EGOT* expression were noted for other clinical parameters in both HPV groups (Table 2).

### 3.2. Patients with Increased Expression of lncRNA EGOT Have a Better Prognosis

**Figure 3:**
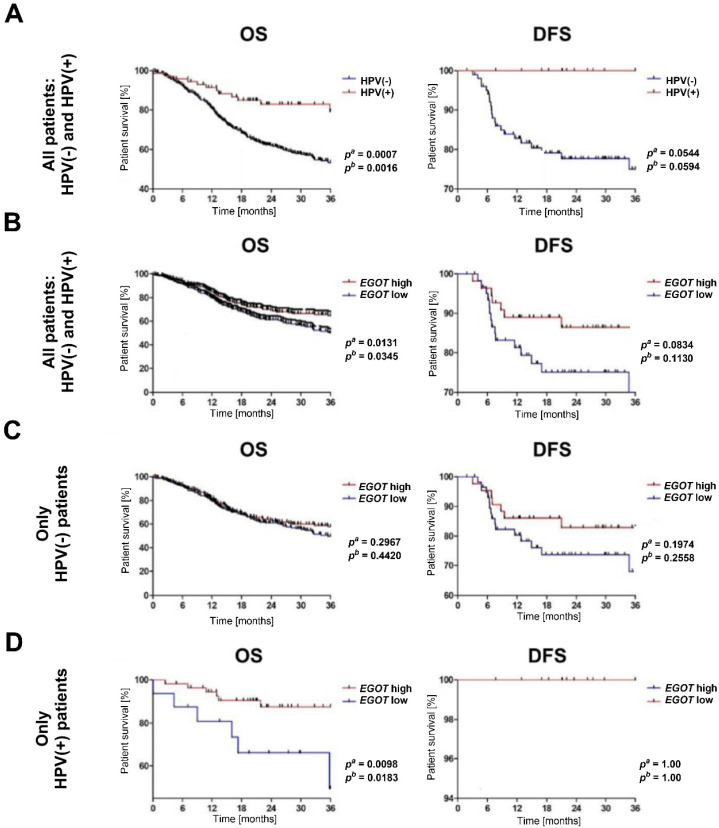
Kaplan–Meier survival analysis in HNSCC patients. (**A**) Overall survival (OS) and disease-free survival (DFS) by HPV status. (**B**) OS and DFS by *EGOT* expression level in the entire cohort (HPV(−) and HPV(+)). (**C**) OS and DFS by *EGOT* expression level in the HPV(−) group. (**D**) OS and DFS by *EGOT* expression level in the HPV(+) group. Statistical significance was evaluated using the log-rank test.

The analysis of overall survival and disease-free survival indicated that HNSCC patients with a HPV(+) profile exhibited significantly longer overall survival OS compared to HPV(−) patients (*p^a^* = 0.0007, *p^b^* = 0.0016). While disease free survival (DFS) differed between the HPV(+) and HPV(−) groups, this difference was not statistically significant (*p^a^* = 0.0544, *p^b^* = 0.0529) (Figure 3A). When stratified by *EGO*T expression levels, patients with high *EGO*T expression demonstrated longer OS (*p^a^* = 0.0131, *p^b^* = 0.0345). Although DFS was also prolonged in patients with high *EGOT* expression, the difference was not statistically significant (*p^a^* = 0.0834, *p^b^* = 0.113); (Figure 3B). Further analysis of the significance of *EGO*T in patient survival was conducted separately within the HPV(−) and HPV(+) subgroups. Among HPV(−) patients, no statistically significant differences in OS and DFS were observed based on *EGOT* expression levels (OS: *p^a^* = 0.2967, *p^b^* = 0.442; DFS: *p^a^* = 0.1974, *p^b^* = 0.2558); (Figure 3C). Conversely, in the HPV (+) subgroup, patients with high *EGOT* expression showed significantly longer OS compared to those with low *EGOT* expression (*p^a^* = 0.0098, *p^b^* = 0.0183), although no statistically significant differences were observed for DFS (Figure 3D).

### 3.3. Expression of lncRNA EGOT Is Associated with Changes in Genes Related to Cell Cycle, Immune Response and Viral Infections

Based on the data collected from the Reactome database, an analysis of genes positively and negatively correlated with *EGOT* expression was performed for further analysis. Genes were included in the analysis if they met the following criteria: a correlation coefficient (R) greater than 0.3 or less than −0.3, and a *p*-value less than 0.05 (Supplementary Table S3). Data are presented as the number of genes from the studied list relative to the total number of possible genes within a given signaling pathway and plotted as graphs.

The associated genes were implicated in various biological processes in the HPV(−) group. Specifically, genes were related to the organization of intercellular connections, with 1 gene out of 94 involved in this process. Nervous system development was associated with 1 gene out of 620. Transcriptional regulation of stem cells involved 1 gene out of 45, while EGFR signal transduction included 1 gene out of 499. Collagen formation was linked to 1 gene out of 104, and extracellular matrix degradation was related to 1 gene out of 148. Interactions with surface integrins were associated with 1 gene out of 86, and gene splicing involved 1 gene out of 117. The innate immunity process included 4 genes out of 1329, and adaptive immunity involved 2 genes out of 999. Cytokine signaling was associated with 6 genes out of 1312. Inositol phosphate metabolism was related to 1 gene out of 90, lipid metabolism involved 2 genes out of 1445, and vitamin metabolism included 1 gene out of 383. Amino acid metabolism was associated with 2 genes out of 662, and carbon dioxide hydration involved 1 gene out of 17. Post-translational modification of proteins included 4 genes out of 1594. Cell signaling was linked to 1 gene out of 352, while apoptosis involved 91 genes out of 128. Necrosis was associated with 1 gene out of 28. Tyrosine receptor signaling included 1 gene out of 622, MAPK signaling was related to 1 gene out of 403, and death receptor signaling involved 1 gene out of 157. Transmembrane transport was associated with 1 gene out of 419, and ion transport involved 1 gene out of 207 (Supplementary Figure S2).

**Figure 4:**
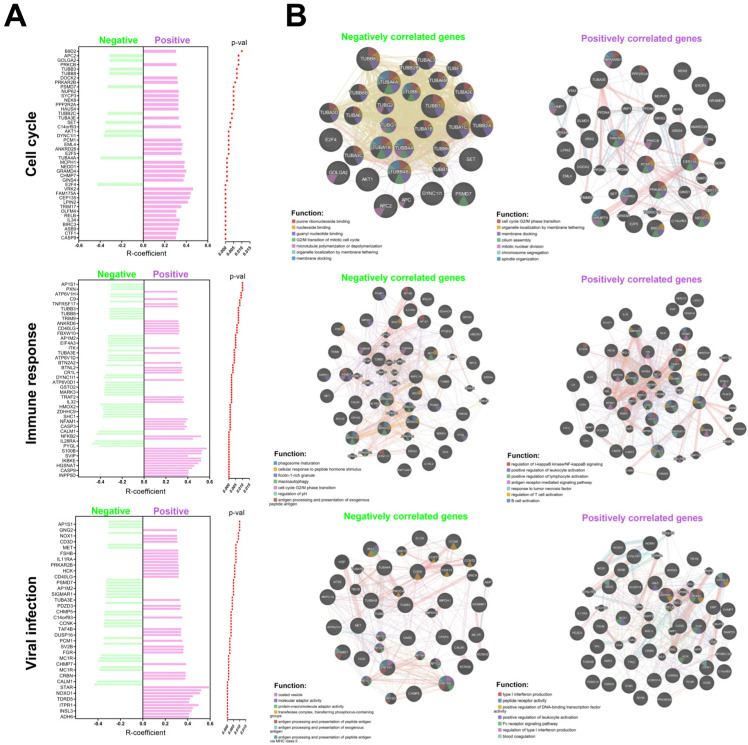
Association of *EGOT* with molecular pathways and cellular processes: (**A**) list of genes correlated with *EGOT* in individual pathways including cell cycle, immune response and connected with viral infections the HPV(−) and HPV(+) groups; and (**B**) function and interaction of positively and negatively correlated genes with *EGOT* for HPV(+) patients. Graphs generated using GeneMANIA online tool included correlated genes with *EGOT* (*p* < 0.01 and R > 0.3 and R < −0.3) and predicted genes during analysis within cell cycle, immune response, and connection with viral infections.

Furthermore, in the HPV(+) group, genes with positive correlations involved in several critical biological processes were also documented. Specifically, these genes are associated with mitosis, with 22 genes out of 596 implicated in this process. Cellular response to stress involves 19 genes out of 691, while chromatin organization includes 16 genes out of 256. The development of the nervous system is associated with 40 genes out of 620, and transcriptional regulation of stem cells involves 13 genes out of 45. Keratinization is linked to 19 genes out of 226, and diseases related to EGFR signal transduction involve 39 genes out of 499. Metabolic diseases are associated with 19 genes out of 409, and infectious diseases involve 58 genes out of 1374. Transcription of RNA polymerase II includes 153 genes out of 1693. The innate immunity process involves 99 genes out of 1329, while acquired immunity includes 102 genes out of 999. Cytokine signaling is associated with 135 genes out of 1312. Metabolism of carbohydrates involves 19 genes out of 456, lipid metabolism includes 66 genes out of 1445, and biological oxidation is linked to 23 genes out of 545. Post-translational protein modification involves 80 genes out of 1594, and RNA metabolism includes 26 genes out of 782. Nervous system processes are associated with 55 genes out of 498, while biogenesis and maintenance of cellular organelles involve 24 genes out of 335. Apoptosis includes 15 genes out of 189, and GPCR signaling involves 93 genes out of 1485. MAPK signaling is associated with 29 genes out of 403, Rho GTPase signaling involves 31 genes out of 457, small molecule transport includes 50 genes out of 963, and vesicular transport involves 44 genes out of 824 (Supplementary Figure S3). Conversely, negatively correlated genes in the HPV(+) group are also associated with several key biological processes. Mitosis involves 11 genes out of 596, while cellular response to stress includes 14 genes out of 691. The development of the nervous system is associated with 13 genes out of 620, and regulation of beta cell development involves 3 genes out of 67. Infectious diseases are linked to 26 genes out of 1374, and RNA polymerase II transcription includes 25 genes out of 1693. Megakaryocyte development involves 9 genes out of 194, while innate immunity includes 18 genes out of 1329. Acquired immunity is associated with 21 genes out of 999, and cytokine signaling involves 23 genes out of 1312. Post-translational protein modification includes 26 genes out of 1594, and RNA metabolism involves 18 genes out of 782. Nuclear receptors are linked to 17 genes out of 385, while death receptor signaling includes 8 genes out of 157. Rho GTPase signaling involves 13 genes out of 457, small molecule transport includes 23 genes out of 963, and vesicular transport involves 21 genes out of 824 (Figure 4 and Supplementary Figure S2).

**Figure 5:**
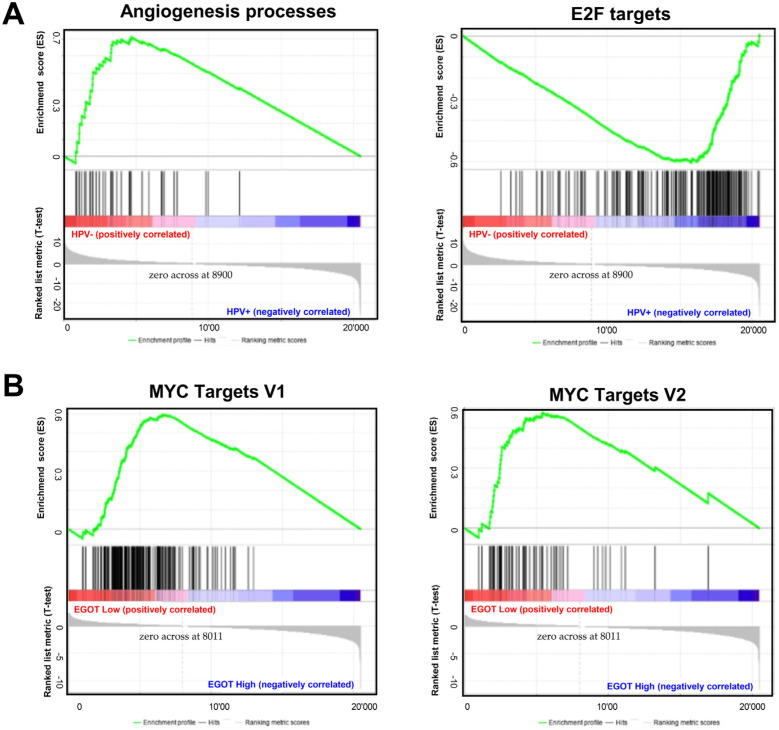
Molecular pathways depending on HPV status and expression levels of *EGOT*: (**A**) Gene Set Enrichment Analysis (GSEA) between HPV(−) and HPV(+) HNSCC patients’ groups and (**B**) in HPV(+) HNSCC patients divided in high and low *EGOT*-expression groups; ES—enrichment score, only results with *p* ≤ 0.05 and *FDR* ≤ 0.25 were shown.

Gene Set Enrichment Analysis (GSEA) between HPV(−) and HPV(+) patient groups revealed that several signaling pathways directly or indirectly related to carcinogenesis are enriched in both groups. In the HPV(−) group, genes responsible for angiogenesis processes were significantly enriched (*p* = 0.0019, *FDR* = 0.209). In the HPV(+) group, genes involved in cell cycle control were significantly enriched (*p* = 0.0273, *FDR* = 0.185); (Figure 5A). When the HPV(−) group was further stratified by *EGOT* expression levels (high/low), a greater number of enriched genes were observed in the group with high *EGOT* expression; however, these results did not reach statistical significance. In contrast, in the HPV(+) group divided by *EGOT* expression levels (high/low), there was a significant enrichment of genes regulated by *MYC-V1* (*p* < 0.0001, *FDR* = 0.0184) and *MYC-V2 (p* = 0.024, *FDR* = 0.036) in the low *EGOT* expression group (Figure 5B).

### 3.4. lncRNA EGOT Interacts with Hsa-miR-3909 Based on Prediction Analysis in HNSCC

To analyze the potential interaction between miRNA and *EGOT*, the ENCORI database was utilized. We selected *hsa-miR-6509-3p*, *hsa-miR-3617-5p*, *hsa-miR-641*, *hsa-miR-33b-5p*, *hsa-miR-33a-5p*, *hsa-miR-6852-3p*, and *hsa-miR-3909* as the miRNAs which could have *EGOT* lncRNA as the potential target based on the base-pairing analysis between miRNA:lncRNA sequence (Table 3).

This analysis led to the selection of seven miRNAs for further in-depth investigation. Spearman’s rank correlation tests were conducted to assess potential correlations between the expression levels of selected miRNAs and *EGOT* within HPV(+) and HPV(−) groups. The analysis revealed a statistically significant correlation for *hsa-miR-3909* in the HPV(−) group (R = −0.3079, *p* = 0.024), which was not observed in the HPV(+) group (R = 0.2706, *p* = 0.134). For both *hsa-miR-3617-5p* and *hsa-miR-6852-3p*, there was insufficient data to perform the correlation test (Table 4).

**Figure 6:**
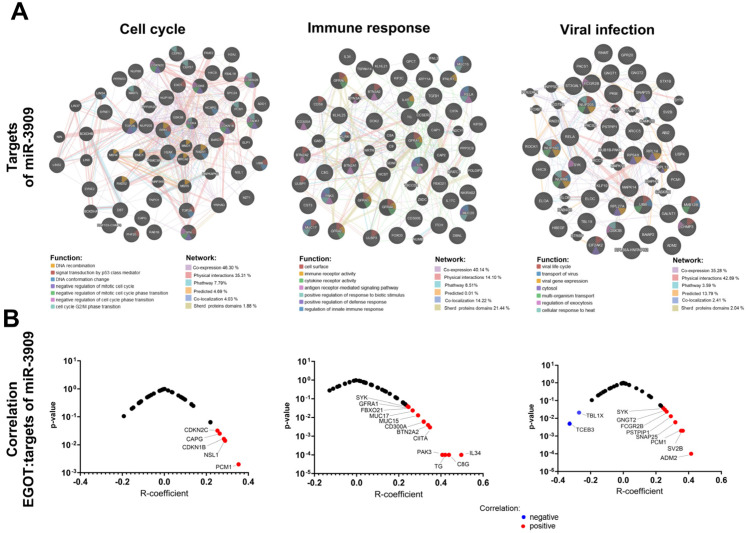
Analysis of the association of lncRNA *EGOT* and *miR-3909*: (**A**) genes related to cell cycle, immune response, and response to viral infection that are regulated by *miR-3909* and (**B**) correlation of lncRNA *EGOT* with *miR-3909* targets.

Given the potential regulation of miRNA expression by the *EGOT* transcript, *hsa-miR-3909* was selected for further analysis. Predictive targets for *hsa-miR-3909* were identified using the TargetScanHuman database and classified into genes connected with the cell cycle (including processes: DNA recombination, signal transduction by *p53* class mediator, DNA conformation change, negative regulation of mitotic cell cycle, negative regulation of mitotic cell cycle phase transition, negative regulation of cell cycle phase transition, and cell cycle G2/M phase transition), immune response (including processes: cell surface, immune receptor activity, cytokine receptor activity, antigen receptor-mediated signaling pathway, positive regulation of response to biotic stimulus, positive regulation of defense response, and regulation of innate immune response) as well as genes connected with viral infection (including processes: viral life cycle, transport of virus, viral gene expression, processes in cytosol, multi-organism transport, regulation of exocytosis, and cellular response to heat) based on the GeneMania tool analysis. The correlation between the expression levels of these target genes and the levels of *hsa-miR-3909* and *EGOT* was examined in the HPV(+) patient group. We indicated that significant correlations were observed for *CAPG* (R = 0.2636; *p* = 0.025), *CDNK1B* (R = 0.2835; *p* = 0.016), *CDNK2C* (R = 0.2529; *p* = 0.032), *NSL1* (R = 0.289; *p* = 0.014), and *PCM1* (R = 0.3535; *p* = 0.002) in the cell cycle, *BTN2A2A* (R = 0.0994; *p* = 0.004), *C8G* (R = 0.438; *p* < 0.0001), *CD300A* (R = 0.3184; *p* = 0.006), *CIITA* (R = 0.3478; *p* = 0.003), *FBOXO21* (R = 0.2463; *p* = 0.037), *GFRA1* (R = 0.2369; *p* = 0.045) *IL34* (R = 0.4965, *p* < 0.0001), *MUC15* (R = 0.2917; *p* = 0.013), *MUC17* (R = 0.2659; *p* = 0.024), *PAK3* (R = 0.4067; *p* < 0.0001), *TG* (R = 0.4204; *p* =< 0.0001), and *SYK* (R = 0.2435; *p* = 0.039) in immune response, *ADM2* (R = 0.415; *p* < 0.0001), *FCGR2B* (R = 0.2652; *p* = 0.024), *GNGT2* (R = 0.2543; *p* = 0.031), *PSTPIP1* (R = 0.2907; *p* = 0.013), *SNAP25* (R = 0.3184; *p* = 0.006), *SV2B* (R = 0.364; *p* = 0.002), *SYK* (R = 0.2435; *p* = 0.039), *TBL1X* (R = ࢤ0.2707; *p* = 0.021), *TCEB3* (R = ࢤ0.3282; *p* = 0.005), and *PCM1* (R = 0.3535; *p* = 0.002) in viral infection processes (Figure 6, and Supplementary Table S3).

## 4. Discussion

During transcription, the human genome creates many different types of non-coding RNAs [32,33,34,35,36] including long non-coding RNAs (lncRNAs), defined as RNA molecules longer than two hundred nucleotides that are not translated into proteins. It is well-known that RNA Polymerase II transcribes most lncRNA; consequently, many possess 5′-end m7G caps and 3′-end poly(A) tails, similar to mRNAs [37]. However, recent research has demonstrated unique features of lncRNA transcription, processing, and sending that are intricately linked to their cellular roles. Compared to mRNAs, a higher proportion of lncRNAs remains in the nucleus, although a major fraction is transported to the cytoplasm [38,39]). These cytosolic long ncRNAs likely share their action and stimulate export pathways with mRNAs. Moreover, lncRNAs regulate gene expression at multiple levels by interacting with DNA, RNA, and proteins; they can modulate chromatin structure and function, influence the transcription of both neighboring and distant genes, and affect RNA splicing, stability, and translation [40,41,42].

In cancer, lncRNAs demonstrate either tumor-suppressive or tumor-promoting roles by influencing cellular pathways involved in cancer initiation, progression, and metastasis. It has been documented that lncRNAs are involved in critical signaling pathways such as *Wnt/β-catenin*, *Hippo*, *Notch*, *NF-κB*, *Hedgehog*, *TGF-β*, *JAK-STAT*, and *PI3K/AKT* [43]. In head and neck squamous cell carcinomas HPV(+) patients, the presence of a viral genome can trigger dysregulation of lncRNAs [32]. Thus, this report highlights the changes in the cell transcriptome induced by viral infection and explores their potential connections to the pathophysiology of HNSCC.

Among ninety selected lncRNAs in HNSCC patients, we observed a statistically significant higher expression in HPV(+) samples in comparison to HPV(−) samples for *EGOA*, *EGOB*, *HAR1B*, and *HOXA-AS2* (*HOXA3as*) and no expression of *GAS5*-family compared to HPV(−) samples.

First of all, the results and conclusions presented in this study are consistent with those reported by us previously. Importantly, the majority of these findings were derived from pre-existing data available in databases such as cBioPortal and UALCAN, rather than from independent experimental analyses. We described that *EGOT* expression levels depend on tumor grading and location. *EGOT* was slightly down-regulated in G1–3 tumors relative to healthy tissue and up-regulated in G4 tumors, and its expression is lower in the oral cavity and larynx but on the other hand, higher in the pharynx [44].

At the time of publishing our previous findings, additional supporting information was not yet available. However, recent data reinforce both our earlier conclusions and the results presented here. Notably, in her PhD dissertation, Swati Tomar analyzed gene expression in oropharyngeal cancers, comparing HPV(+) and HPV(−) patient groups. One of the dissertation appendices includes an analysis of lncRNA EGOT expression levels, revealing its upregulation in HPV-active vs. HPV-inactive (3.01- and 4.35-fold changes) and HPV-active vs. HPV(+) (2.84- and 4.22-fold changes) patients. Conversely, EGOT was downregulated in HPV-inactive vs. HPV(−) groups (1.06- and 1.03-fold changes) [45]. These findings suggest that EGOT expression is upregulated in HPV(+) patients, with a potential role in the early stages of viral infection. This may explain why some patient groups exhibit little to no change in *EGOT* expression. It should be noted that Tomar’s study utilized different patient cohorts and methodologies than those presented here. Additionally, S. Tomar research primarily focused on protein-coding genes rather than lncRNAs. Our observations align with other studies linking lncRNA *EGOT* to viral-related diseases. For instance, *EGOT* expression is disrupted in COVID-19 patients, with higher levels detected in individuals with moderate symptoms compared to asymptomatic cases. Moreover, *EGOT* expression was positively correlated with NRAV (negative regulator of antiviral response) levels, particularly in asymptomatic patients [46]. These findings suggest that *EGOT* is upregulated during the early stages of viral infections but declines as the infection progresses. Furthermore, Carneiro et al. demonstrated that hepatitis C virus (HCV) infection requires elevated *EGOT* expression, with lower *EGOT* levels associated with reduced viral genome levels. Their study also revealed that *EGOT* expression can be modulated by antiviral drugs such as sofosbuvir, daclatasvir, and ribavirin, as well as by dsRNA, synthetic analogs, and viral RNA. Importantly, *EGOT* is regulated by *NF-κB*, which binds to its promoter [47].

Dysregulated *EGOT* expression has been observed in various cancers, including head and neck [44,48], breast [49,50,51,52], hepatocellular carcinoma [53], rectal cancer [54], gastric cancer [55], renal cell carcinoma [56,57], thyroid cancer [58,59,60,61], and glioma [62]. While these cancers may initially appear unrelated, certain subtypes share links to viral carcinogenesis, such as HNSCC [23], hepatocellular carcinoma [63], and colorectal cancer [64], or other pathogens, as in the case of *Helicobacter pylori*-associated gastric cancer [65]. Additionally, some studies suggest a potential role for viral infections in the pathogenesis of breast and renal cancers [66,67]. Another shared characteristic among these cancers is their varying degrees of immunogenicity, which applies to HNSCC, breast cancer, hepatocellular carcinoma, rectal cancer, colorectal cancer, gastric cancer, renal cell carcinoma, thyroid cancer, and glioma [68,69,70,71,72]. Given that patient survival is a crucial clinical parameter, identifying reliable prognostic biomarkers remains a major research focus. Our findings indicate that lncRNA *EGOT* could serve as a potential prognostic biomarker for overall survival. Interestingly, this association was observed in both the full patient cohort (HPV(+) and HPV(−)) and in HPV(+) patients alone. Surprisingly, no significant correlation was found between *EGOT* expression levels and OS in the HPV(−) patient group. Moreover, overall survival curves and disease-free survival time analyses showed that HPV(+) patients had significantly longer OS. However, based on the literature and clinical point of view, This observation is in line with the study published by Nichols et al., where HPV(+) patients with oropharyngeal cancers exhibited longer OS and DFS compared to HPV(−) patients [73]. Furthermore, HPV(+) patients with metastatic oropharyngeal cancer also demonstrated better OS in the Zhou et al. study [74]. In previous studies, lncRNA *EGOT* has been identified as a potential prognostic biomarker in various cancers. Lower *EGOT* expression has been associated with improved survival in rectal [54], gastric [55], and colon cancers [75], while higher expression correlates with poorer outcomes in breast cancer [49]. However, when included in a panel of lncRNAs, higher *EGOT* expression was linked to a low-risk group of breast cancer patients with better survival [51]. Carnero et al. suggested that *EGOT* promotes viral replication by suppressing the interferon (*IFN*)-mediated antiviral response [47]. Our analysis revealed correlations between *EGOT* expression and genes involved in the cell cycle, immune response, and viral infections. Current knowledge indicates that lncRNA *EGOT* is upregulated in response to cellular stress, including pathogen exposure, through alterations in the *PI3K/AKT*, *MAPK*, and *NF-κB* pathways [76], contributing to cell survival and inflammation. One key gene family involved is interferon-stimulated genes (ISGs), which regulate viral replication and apoptosis [77].

To further investigate, we analyzed genes positively correlated with lncRNA *EGOT* in HPV(+) patients, focusing on genes differentially expressed in HPV-Active vs. HPV-inactive groups. Our findings revealed that genes associated with the cell cycle (*GINS4*, *GRAMD4*, *NEDD1*, *NUP62*), immune response (*IKBKE*, *IL34*, *NFKB2*, *PLAC8*, *RELB*, *SVIP*, *TRAF2*, *VCAM1*), and viral infection (*ANKRD6*, *APOBEC3G*, *DBP*, *IL11RA*, *STAR*, *SV2B*, *TDRD10*, *TYK2*, *VHL*) were upregulated, consistent with Tomar’s microarray analysis [45]. Using the UALCAN database, we confirmed significant upregulation of these genes in HPV(+) *versus* HPV(−) head and neck squamous cell carcinoma patients, underscoring the role of *EGOT* in HPV infection. Another group of genes correlated with *EGOT* was linked to immune response regulation. Zhao et al. demonstrated that *EGOT* is among the immune-related lncRNAs implicated in periodontal tissue inflammation. Their study on gingival fibroblasts with *EGOT* knockdown and TNF stimulation showed decreased *IL-1β*, *IL-6*, and *IL-8* expression, regulated via the NF-κB pathway [78]. We observed a correlation between *EGOT* and *IL-34* in both HPV(+) and HPV(−) patients, with Tomar’s study also reporting *IL-34* upregulation in HPV-active and HPV-inactive vs. HPV(+) groups. Additionally, *IL11RA* was upregulated exclusively in HPV(+) patients and correlated with *EGOT* expression, aligning with Tomar’s findings in HPV-active patients. Analysis of breast cancer samples from the TCGA data and qRT-PCR studies suggested that lncRNAs, including *EGOT*, could serve as independent prognostic markers [51,52]. These genes were categorized as genomic instability-related lncRNAs associated with immune checkpoint regulation. Patients classified as high-risk by this model exhibited immune checkpoint disturbances, increased DNA replication, enhanced cell cycle progression, and tryptophan metabolism dysregulation, resulting in poorer survival. The authors proposed that lncRNA analysis could aid in pharmacogenomics, particularly in predicting responses to immune checkpoint inhibitors (ICIs) in breast cancer patients [51,52]. Our findings also identified immune-related genes correlated with *EGOT*, warranting further exploration of its role in immune regulation.

Additionally, lncRNA *EGOT* has been implicated in the response to irradiation [54]. Surgery, radiotherapy, and chemotherapy remain primary treatment modalities for HNSCC [79,80]. Given the need to mitigate treatment-related complications such as oropharyngeal dysphagia [3], radiation dermatitis [81], brachial plexopathy [82], and challenges in surgical margin irradiation [83], personalized radiotherapy approaches and targeted therapies utilizing nanodelivery systems for radiosensitization are crucial [84,85,86,87]. Li et al. demonstrated that, in rectal cancer, *EGOT* regulates radiosensitivity via *miR-211-5p* and *ErbB4* expression. *EGOT* expression was elevated in rectal cancer patients and cell lines, correlating with worse survival. Knockdown of *EGOT* reduced proliferation and migration while enhancing radiosensitivity. In vivo experiments in nude mice further confirmed that tumors with reduced *EGOT* expression exhibited the smallest volumes post-irradiation [54]. The study by Wang et al. linked *EGOT* to radiosensitivity through autophagy regulation. The authors reported a negative correlation between *EGOT* and autophagy-related genes *ATG7*, *ATG16L1*, and *LC3II* in renal tubular cells, suggesting that the *HuR/EGOT/ATG7/16L1* axis is essential for autophagy under hypoxic conditions [56]. To examine *EGOT’*s potential role in radioresistance in HNSCC, we analyzed its correlations with *ErbB4*, *miR-211-5p*, *HuR* (*ELAVL1*), *ATG7*, *ATG16L1*, and *LC3II* (*MAP1LC3B*) in HPV(+) and HPV(−) patients, as well as in the entire cohort. However, no significant associations were observed. Furthermore, ENCORI database analysis did not reveal strong correlations between *EGOT* and the genes described by Li et al. and Wang et al. in HNSCC patients. While *EGOT* correlated positively and negatively with various cell cycle genes, none were linked to irradiation response, highlighting the need for further investigation. Finally, we explored *EGOT’*s interactions with miRNAs using base-pairing analysis from the ENCORI database. We identified *hsa-miR-6509-3p*, *hsa-miR-3617-5p*, *hsa-miR-641*, *hsa-miR-33b-5p*, *hsa-miR-33a-5p*, *hsa-miR-6852-3p*, and *hsa-miR-3909* as potential candidates. Of these, only *hsa-miR-3909* exhibited a significant negative correlation with EGOT. Notably, previously reported interactions with *miR-221-5p* [54], and *miR-641* [58] were not observed in our dataset, suggesting that *EGOT*-miRNA interactions may be context-dependent.

In conclusion, our findings emphasize the multifaceted role of lncRNA *EGOT* in cancer progression, immune response, viral infection, and radiosensitivity. While previous studies suggest *EGOT’*s involvement in autophagy and radiation response, our results did not confirm these associations in HNSCC. However, *EGOT*’s correlation with immune and cell cycle genes, along with its potential interaction with miRNAs, warrants further research to elucidate its precise mechanistic contributions and potential as a therapeutic target.

Our analysis of the correlation between miRNAs and *EGOT* revealed a significant positive correlation between the expression of *hsa-miR-6509-3p* and *EGOT*. However, due to the potential regulation of miRNA expression levels by the *EGOT* transcript, *hsa-miR-3909* was chosen for further analysis. Correlation analysis of *hsa-miR-3909* showed that its expression was associated with increased expression of many genes involved in processes such as the cell cycle, immune response, and viral infection. Interestingly, the only genes negatively correlated with *hsa-miR-6509-3p* were *TBL1X* and *TCEB3*. The correlation analysis highlighted that *TYMS* and *PDCD1* were positively correlated with *EGOT* expression only in the HPV(+) group, while *ITPR1* showed a positive correlation in both HPV groups, with a stronger correlation in the HPV(+) group. In HPV(+) HNSCC patients, higher *TYMS* expression correlates with a poorer response to 5-FU treatment than was noted in the case of colorectal cancer [88]. In the context of our report given the increased expression of *EGOT* in HPV(+) HNSCC patients, it can be inferred that high *EGOT* expression may indicate resistance to 5-FU therapy.

It is known that *PDCD1* encodes the programmed cell death protein 1 (PD1), a target of the anti-PD1 drug pembrolizumab, which binds to PD1 and may stimulate a cytotoxic response from Tc cells. In our study, the positive correlation between *PDCD1* and *EGOT* suggests an enhanced response to pembrolizumab. While clinical trials have indicated a synergistic effect of cetuximab plus a PD-1 inhibitor compared to PD-1 inhibitor monotherapy in recurrent or metastatic HNSCC, Zhang et al.’s meta-analysis reveals that this combination therapy is likely effective in improving response rate and 1-year overall survival only in HPV(−) patients [89].

Widely known Inositol 1,4,5-trisphosphate receptor type 1 (ITPR1) is part of a channel that regulates calcium ion flow into cells. The analysis showed that *ITPR1* expression correlated positively with *EGOT* in both HPV(−) and HPV(+) groups, with a higher correlation in the HPV(+) group. This finding is consistent with the study by Xu et al., which demonstrated that *EGOT* increases autophagosome accumulation by upregulating *ITPR1* expression, thereby sensitizing cells to paclitaxel toxicity. *EGOT* upregulates *ITPR1* levels by generating *pre-ITPR1/EGOT* dsRNA, which induces *pre-ITPR1* accumulation to increase *ITPR1* protein expression in cis, and by recruiting *hnRNPH1* to enhance *pre-ITPR1* alternative splicing in trans [90].

In this work, we also analyzed the phenotype of patients based on HPV status (HPV(+) and HPV(−) groups) and *EGOT* expression using the GSEA tool, which compares the analyzed groups with known gene expression profiles from specific pathways or processes. In the HPV(−) group, genes responsible for angiogenesis were enriched. In HNSCCs, angiogenesis promoters, such as interleukin-8, are associated with poor survival [91]. In the HPV(+) group, genes involved in the cell cycle control and targeted by *E2F* were enriched, a result expected due to the degradation of Rb proteins by the HPV E7 protein. Further, in the HPV(+) group, there was an enrichment of genes regulated by the *MYC* family, which are proto-oncogenes encoding other transcription factors. In many cancers, *c-myc* is constitutively expressed, leading to increased expression of genes responsible for cell proliferation, contributing to tumor formation [92]. Although *MYC* amplification is associated with the development of HNSCC, analyses have shown no prognostic significance and no association with HPV infection. However, HPV E6 and E7 knockdown causes a drastic decrease in *MYC* expression, potentially leading to reduced viability of cervical cancer cells [93].

Given the above results, it can be assumed that *EGOT* expression testing may serve as an additional marker to distinguish HPV(−) from HPV(+) samples in HNSCCs, as this gene is significantly overexpressed in HPV(+) tissues. The patients with high *EGOT* expression also demonstrate significantly longer OS, suggesting its potential use as a prognostic factor. The positive correlation of *TYMS*, *PDCD1*, and *ITPR1* genes with *EGOT* in HPV(+) samples could guide selection of the most effective therapy. Furthermore, the enriched signaling pathways in patients with increased *EGOT* expression in the HPV(+) group could serve as reference points for targeted therapies.

There are certain limitations to our study. First, our cohort was relatively small in the in vitro portion of the study. Additionally, some clinical data were not available for the in silico analysis. Despite these limitations, overall, our findings shed light on incorporating *EGOT* expression analysis in clinical practice to enhance patient stratification and treatment efficacy in HNSCC.

We hypothesize that changes in *EGOT* expression represent a universal host cell response to viral entry. This hypothesis is supported by the observed upregulation of lncRNA *EGOT* across various viral-mediated diseases, both cancerous and non-cancerous. Upregulation has been reported in infections caused by SARS-CoV-2 (severe acute respiratory syndrome coronavirus 2; (+)ssRNA) [46], IAV (influenza A virus; (−)ssRNA), and SMV (Semliki Forest virus; (+)ssRNA) [93], as well as in viruses linked to benign lesions and cancer, such as HPV (human papillomavirus; DNA virus) [45] and HCV (hepatitis C virus; (+)ssRNA) [47]. As proposed by Carnero et al. [47], we suggest that lncRNA *EGOT* contributes to the host’s antiviral response by modulating the transcriptome and altering immune pathways that influence viral replication. However, further studies are still required to confirm the role of lncRNA EGOT in HPV viral infection and controlling immune response and verify the molecular mechanisms involved in HNSCC.

## Figures and Tables

**Table 1:** Patient characteristics enrolled in in vitro analyses: NA—data not-available, NEG—negative, POS—positive.

Patient ID	Gender	Age at Diagnosis	Tumour Location	TNM	Grade	HPV Status
P1	M	63	Floor of mouth/tongue	T4N3M0	G2	NEG
P2	M	80	Oral vestibule	T2N0M0	G2	NEG
P3	M	59	Pharynx/tongue	NA	G2	NEG
P4	M	68	Tongue	T3N2M0	G3	NEG
P5	M	59	Glottis	T2N0M0	NA	NEG
P6	M	67	Larynx	T2N0M0	NA	NEG
P7	M	56	Larynx	NA	NA	NEG
P8	M	61	Pharynx	T2N0M0	G3	NEG
P9	M	66	Larynx	T3N0M0	G2	NEG
P10	M	63	Tongue	T4N3M0	G2	NEG
P11	F	55	Glottis	T2N0M0	NA	NEG
P12	M	63	NA	NA	NA	POS
P13	F	42	Tonsil	T1N2M0	NA	POS
P14	M	68	Floor of mouth/tongue	NA	NA	NEG

**Table 2:** *EGOT* expression level depending on clinicopathological parameters in HPV(+) and HPV(−) groups of HNSCC; *t*-test or Mann–Whitney U test; *p* < 0.05 considered significant.

Patients		HPV(+)		HPV(−)	
Parameter	Group	Mean ± SEM	*p*-Value	Mean ± SEM	*p*-Value
Age	<60	3.184 ± 0.1959	0.011	1.655 ± 0.07591	0.261
	≥60	2.337 ± 0.2445		1.540 ± 0.06746	
Gender	Woman	3.116 ± 0.8720	0.703	1.564 ± 0.09399	0.767
	Man	2.873 ± 0.1613		1.597 ± 0.05999	
Alcohol consumption	Yes	3.003 ± 0.1805	0.152	1.600 ± 0.06063	0.625
	No	2.423 ± 0.3240		1.547 ± 0.09402	
Smoking	Yes	3.768 ± 0.3190	0.529	1.621 ± 0.06195	0.389
	No	3.061 ± 0.2912		1.531 ± 0.08673	
Cancer stage	I + II	2.962 ± 0.4007	0.842	1.619 ± 0.09422	0.847
	III + IV	2.875 ± 0.1757		1.595 ± 0.06031	
Tumour size	T1 + T2	3.193 ± 0.2196	0.049	1.602 ± 0.08685	0.987
	T3 + T4	2.558 ± 0.2256		1.600 ± 0.06328	
Nodal status	N0	2.523 ± 0.2735	0.16	1.651 ± 0.06863	0.395
	N1 + N2 + N3	3.006 ± 0.1921		1.563 ± 0.07850	
Cancer grade	G1 + G2	2.786 ± 0.2473	0.85	1.540 ± 0.05488	0.192
	G3 + G4	2.852 ± 0.2443		1.702 ± 0.1266	
Perineural invasion	Yes	3.460 ± 0.5076	0.12	1.605 ± 0.08431	0.873
	No	2.512 ± 0.2549		1.585 ± 0.08880	
Lymphovascular invasion presence	Yes	2.676 ± 0.2288	0.349	1.596 ± 0.05518	0.916
	No	3.071 ± 0.3143		1.576 ± 0.1649	

**Table 3:** Interaction between *EGOT* sequence and selected miRNAs.

miRNA	lncRNA	Start Point	End Point	Interaction Type	miRNA Sequence	Spot of Interaction	lncRNA Sequence
*hsa-miR-6509-3p*	*EGOT*	4791222	4791243	8mer	uuUAAUC-CAUCACCGUCACCUu	:| || | ||| ||||||||	guGUCAGUGAAGU-GCAGUGGAa
*hsa-miR-3617-5p*	*EGOT*	4791252	4791271	7mer-m8	ggguagaACGUUGAUACAGAAa	||||| |||||||	uuuccaaUGCAA--AUGUCUUc
*hsa-miR-641*	*EGOT*	4791252	4791276	7mer-m8	cuccacugaGAUA-GGAUACAGAAa	| || | |||||||	cgaaguuucCAAUGCAAAUGUCUUc
*hsa-miR-33b-5p*	*EGOT*	4791260	4791282	8mer	cgUUACGUUGUC----GUUACGUg	|||||:| || |||||||	auAAUGCGA-AGUUUCCAAUGCAa
*hsa-miR-33a-5p*	*EGOT*	4791260	4791283	8mer	acGUUACGUU---GAUGUUACGUg	:|||||:| :| |||||||	gaUAAUGCGAAGUUUCCAAUGCAa
*hsa-miR-6852-3p*	*EGOT*	4791292	4791310	7mer-m8	gacucCUUG--UCUCCUGu	|||| |||||||	ggcuuGAACUGAGAGGACu
*hsa-miR-3909*	*EGOT*	4791292	4791314	7mer-m8	ucugaCGUCCGGGA-UCUCCUGu	|| | || |||||||	gugugGCUUGAACUGAGAGGACu

**Table 4:** Correlation between *EGOT* expression and expression of selected miRNAs depending on HPV status in HNSCC patients.

Patients’ Group	HPV(−)		HPV(+)	
miRNA	R	*p*-Value	R	*p*-Value
*hsa-miR-6509-3p*	−0.3034	0.932	−0.4928	0.215
*hsa-miR-3617-5p*	NA	NA	NA	NA
*hsa-miR-641*	−0.1622	0.507	−0.0585	0.723
*hsa-miR-33a-5p*	−0.0103	0.932	−0.027	0.87
*hsa-miR-33b-5p*	−0.0431	0.729	−0.0585	0.723
*hsa-miR-6852-3p*	NA	NA	NA	NA
*hsa-miR-3909*	−0.3079	0.024	−0.2706	0.134

## Data Availability

The original contributions presented in this study are included in the article and Supplementary Material. Further inquiries can be directed to the corresponding author.

## Supplementary Materials

The following supporting information can be downloaded at: https://www.mdpi.com/article/10.3390/biomedicines13040798/s1, Table S1: Long non-coding RNAs (lncRNAs) and housekeeping genes analyzed in the study. Figure S1: Distribution of HPV-Positive and HPV-Negative patients by *EGOT* expression levels. Fisher’s exact test. Table S2: List of genes correlated with *EGOT* in individual pathways for the HPV(−) and HPV(+) groups. Table S3: List of genes correlated with *EGOT* in individual pathways including cell cycle, immune response and connected with viral infections the HPV(−) and HPV(+) groups. Figure S2: The Reactome graphical map illustrates the known biological processes and pathways involving *EGOT* among HPV-negative patients. Significant associations are highlighted with yellow lines; Spearman correlation with a *p* < 0.05. Figure S3: The Reactome graphical map illustrates the known biological processes and pathways involving *EGOT* among HPV-positive patients. Significant associations are highlighted with yellow lines; Spearman correlations (positive, A; negative, B) with a *p* < 0.05.
