# Supplementary material for: lncRNA EGOT Is the Marker of HPV Infection and a Prognostic Factor for HNSCC Patients
Source: Biomedicines. 2025 Mar 26;13(4):798. doi: 10.3390/biomedicines13040798 (PMC12025276; doi:10.3390/biomedicines13040798)
Supplement: Supplementary file 1 [file biomedicines-13-00798-s001.zip › biomedicines-3457830-supplementary.pdf]

Suppl. Table S1. Long non-coding RNAs (lncRNAs) and housekeeping genes analyzed in the study.

**lncRNA**	21A, 7SK, 7SL, Air, AK023948, Alpha 280, Alpha 250, ANRIL, anti-NOS2A, antiPeg11, BACE1AS, BC200, CAR Intergenic 10, DHFR upstream transcripts, Dio3os, DISC2, DLG2AS, E2F4 antisense, EgoA, EgoB, Emx2os, Evf1 and Evf2, GAS5-family, Gomafu, H19, H19 antisense, H19 upstream conserved 1&2, HAR1A, HAR1B, HOTAIR, HOTAIR1M, HOTTIP, Hoxa11as, HOXA3as, HOXA6as, HULC, IGF2AS, IPW, Jpx, Kcnq1ot1, KRASP1, L1PA16, p21, RoR, SFMBT2, VLDLR, LOC285194, LUST, Malat1, mascRNA, MEG3, MEG9, MER11C, ncR-uPAR, NDM29, NEAT1, Nespas, NRON, NTT, p53 mRNA, PCGEM1, PR antisense transcripts, PRINS, PSF inhibiting RNA, PTENP1, RNCR3, SAF, SCA8, snaR, SNHG1, SNHG3, SNHG4, SNHG5, SNHG6, Sox2ot, SRA, ST7OT, TEA ncRNAs, Tmevpg1, TncRNA, Tsix, TUG1, UCA1, UM9-5, WT1-AS, Xist, Y RNA-1, Zeb2NAT, Zfas1, Zfhx2as
**housekeeping genes**	18 S rRNA, RNU43, GAPDH, LAMIN A/C, U6

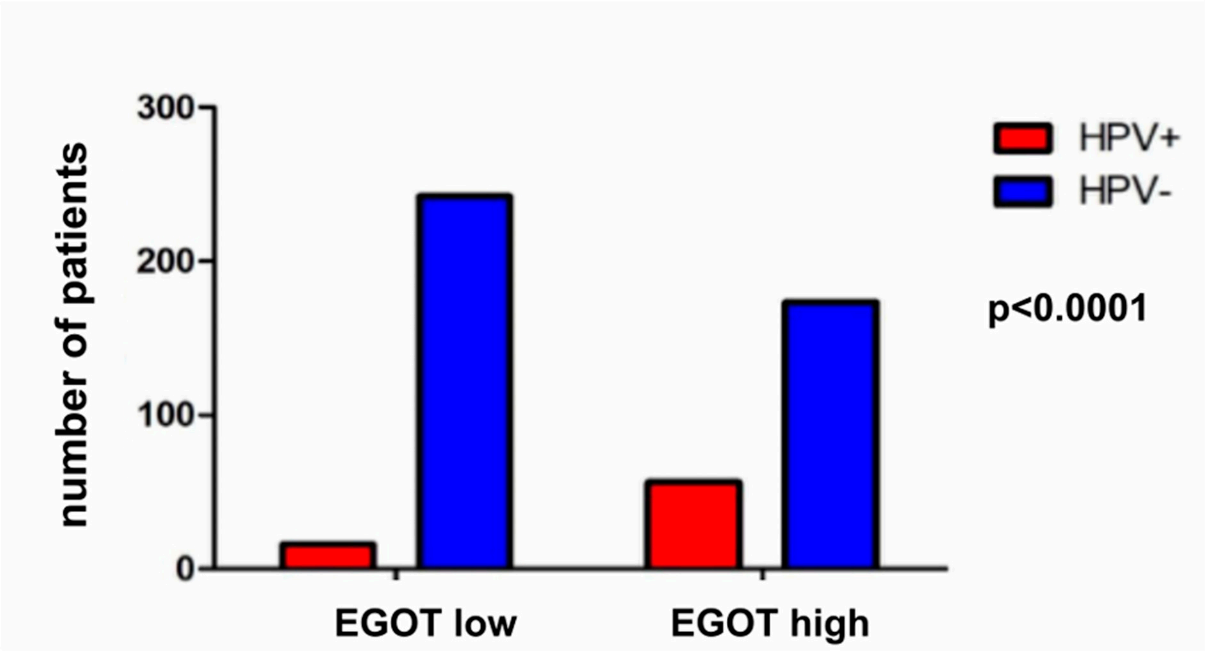

Suppl. Figure S1.Distribution of HPV-Positive and HPV-Negative patients by EGOT expression levels. Fisher's exact test.

Suppl. Table S2. List of genes correlated with EGOT in individual pathways for the HPV(-) and HPV(+) groups.

Role	Gene	R	p	Role	Gene	R	p	Role	Gene	R	p
Cell cycle	ADD1	0.1291	0.28	Immune response	ATP11A	-0.0035	0.977	Viral infection	ABI2	0.036	0.764
	BARD1	0.0851	0.477		BTN2A1	0.1391	0.244		ADM2	0.415	<0.000
	CAPG	0.2636	0.025		BTN2A2	0.339	0.004		BAIAP2	-0.0026	0.983
	CDKN1B	0.2835	0.016		BTN3A2	0.0994	0.406		CHMP3	NA	NA
	CDKN2B	0.0987	0.409		C8G	0.438	0.0001		EIF2AK2	-0.1498	0.209
	CDKN2C	0.2529	0.032		CAP1	-0.1283	0.283		FCGR2B	0.2652	0.024
	CEP57	0.0978	0.414		CD300A	0.3184	0.006		GALNT1	-0.1249	0.296
	CEP63	0.0672	0.575		CD300E	-0.0823	0.492		GNGT1	-0.0845	0.48
	DBT	0.0386	0.747		CD59	0.0677	0.572		GNGT2	0.2543	0.031
	EXO1	-0.0757	0.527		CIITA	0.3478	0.003		GPR20	0.0151	0.9
	FBXL18	-0.0228	0.85		CST3	-0.0553	0.644		HBEGF	-0.1368	0.252
	GSK3B	-0.1213	0.31		DBNL	0.0724	0.545		MAPK14	0.0059	0.961
	H2AFJ	-0.1476	0.216		DOK2	0.1846	0.121		MVB12B	NA	NA
	H2AFX	-0.0185	0.877		FBXO21	0.2463	0.037		PACS1	-0.0749	0.532
	HIST1H4I	-0.003	0.98		FOXO3	0.1213	0.31		PKM	NA	NA
	LIN52	0.1381	0.247		GFRA1	0.2369	0.045		PSTPIP1	0.2907	0.013
	MASTL	0.0178	0.882		GFRA2	0.0432	0.719		RELA	0.2312	0.051
	MZT1	NA	NA		IFNLR1	NA	NA		RNMT	0.1698	0.154
	NCAPG	0.0785	0.512		IL17C	-0.0132	0.912		ROCK1	0.0799	0.505
	NSL1	0.289	0.014		IL34	0.4965	0.0001		RPL14	-0.0397	0.74

	NUP160	-0.0106	0.929		IL4R	0.1015	0.396		RPL27A	0.0007	0.995
	NUP205	-0.1547	0.195		ITCH	0.1573	0.187		RPL36A-HNRNPH2	NA	NA
	NUP88	-0.1928	0.105		KIF3C	0.0329	0.784		RPS4X	-0.005	0.967
	PCM1	0.3535	0.002		KIF5B	0.019	0.874		SNAP25	0.3184	0.006
	PHF20	0.2196	0.064		KLHL21	0.0363	0.762		ST3GAL1	0.157	0.188
	PPP6R3	NA	NA		KLHL25	0.2218	0.061		STX1B	0.2266	0.056
	RAB1B	-0.1169	0.328		MUC15	0.2917	0.013		SV2B	0.364	0.002
	RAD51	-0.115	0.336		MUC17	0.2659	0.024		SYK	0.2435	0.039
	RNF103-CHMP3	NA	NA		MUC20	0.2198	0.064		TBL1X	-0.2707	0.021
	RPA1	-0.0501	0.676		NKIRAS2	0.0119	0.921		TCEB1	-0.0565	0.637
	SFN	-0.0621	0.604		PAK3	0.4067	0.0001		TCEB3	-0.3282	0.005
	SPC24	-0.0111	0.926		PPP3CB	0.1914	0.107		USP4	0.1153	0.335
	SYNE1	0.0902	0.451		QPCT	0.1323	0.268		XRCC5	0.1633	0.17
	SYNE2	0.0012	0.992		TG	0.4204	0.0001		GSK3B	-0.1213	0.31
	TAOK1	0.0351	0.77		TGFB1	-0.0337	0.779		HIST1H4I	-0.003	0.98
	TNPO1	-0.0056	0.962		TSPAN14	0.1917	0.107		NUP160	-0.0106	0.929
	TOP2A	0.1302	0.276		ULBP1	-0.0912	0.446		NUP205	-0.1547	0.195
	TOP3A	-0.0709	0.554		ULBP3	-0.1113	0.352		NUP88	-0.1928	0.105
	UBB	-0.1105	0.355		RELA	0.2312	0.051		PCM1	0.3535	0.002
	YWHAQ	-0.082	0.494		SYK	0.2435	0.039		UBB	-0.1105	0.355

Suppl. Table S3. List of genes correlated with EGOT in individual pathways including cell cycle, immune response and connected with viral infections the HPV(-) and HPV(+) groups.

	HPV-			HPV+		
Role	**Gene**	**R**	**p**	**Gene**	**R**	**p**
Cell cycle	CASP9	0.3038	<0.0001	LPIN2	0.4145	<0.0001
	CTF1	0.3082	<0.0001	CEP135	0.4351	<0.0001
	ASB9	0.3245	<0.0001	FAM175A	0.4412	<0.0001
	BIRC3	0.3331	<0.0001	VRK2	0.4627	<0.0001
	IL34	0.339	<0.0001	E2F4	-0.4261	<0.0001
	RELB	0.3063	<0.0001	GINS4	0.3752	0.001
	OLFM4	0.3092	<0.0001	CHMP7	0.3863	0.001
	TRIM17	0.3532	<0.0001	GRAMD4	0.3997	0.001
				NEDD1	0.3804	0.001
				MCPH1	0.385	0.001
				TUBA4A	-0.3975	0.001
				E2F5	0.356	0.002
				ANKRD28	0.3583	0.002
				EML4	0.3654	0.002
				PCM1	0.3535	0.002
				DYNC1I1	-0.3671	0.002
				AKT1	-0.3539	0.002
				C14orf93	0.3502	0.003

	HPV-			HPV+		
Role	**Gene**	**R**	**p**	**Gene**	**R**	**p**
Immune response				INPP5D	0.4046	<0.0001
				FBXO15	0.4076	<0.0001
				CASP9	0.4086	<0.0001
				C8G	0.438	<0.0001
				HGSNAT	0.4604	<0.0001
				IL34	0.4965	<0.0001
				IKBKE	0.5204	<0.0001
				FCER1A	0.5215	<0.0001
				SVIP	0.4	<0.0001
				VCAM1	0.525	<0.0001
				S100B	0.5292	<0.0001
				RELB	0.5734	<0.0001
				PYGL	-0.4068	<0.0001
				ERP44	-0.4764	<0.0001
				IL28RA	-0.4526	<0.0001
				JAK3	0.4467	<0.0001
				NFKB2	0.5205	<0.0001
				ARPC1A	-0.4327	<0.0001

		SET	-0.3505	0.003			CALM1	-0.4106	<0.0001
		TUBA3E	0.3314	0.004			CRTAM	0.3722	0.001
		TUBB2C	-0.3324	0.004			CASP3	0.3902	0.001
		HAUS4	0.328	0.005			RCHY1	0.3685	0.001
		PPP2R2A	0.3269	0.005			NFAM1	0.3838	0.001
		NEK8	0.3282	0.005			TANK	0.396	0.001
		SYCP3	0.3289	0.005			SHC1	-0.3765	0.001
		NUP62	0.3307	0.005			PDAP1	-0.3713	0.001
		PSMD7	-0.3308	0.005			ZDHHC9	-0.3682	0.001
		PRKAR2B	0.3217	0.006			EDA	-0.3782	0.001
		DOCK2	0.317	0.007			HMOX2	-0.3715	0.001
		TUBB8	-0.3169	0.007			TUBA4A	-0.3975	0.001
		TUBB3	-0.3151	0.007			IL32	0.3645	0.002
		PRKCB	0.3098	0.008			LRRC7	0.3521	0.002
		GOLGA2	-0.3122	0.008			TRAF2	0.3646	0.002
		APC2	-0.3118	0.008			PGAM1	-0.3631	0.002
		B9D2	0.3035	0.010			MARK3	-0.3625	0.002
		ADH6	0.411	<0.0001			IL1RL2	-0.3631	0.002
		ADM2	0.415	<0.0001			GSTO2	-0.3608	0.002
**Viral infection**		INSL3	0.4344	<0.0001			BOLA2	-0.3596	0.002
		JAK3	0.4467	<0.0001			ATP6V0D1	-0.3528	0.002
		ITPR1	0.4963	<0.0001			NFKB1	0.3627	0.002

		NMT2	0.4136	<0.0001
		TDRD5	0.4196	<0.0001
		TYK2	0.4467	<0.0001
		NOXO1	0.5097	<0.0001
		NFKB2	0.5205	<0.0001
		STAR	0.5893	<0.0001
		ARPC1A	-0.4327	<0.0001
		CALM1	-0.4106	<0.0001
		ADRB3	0.3778	0.001
		CRBN	0.3835	0.001
		NCK1	0.3827	0.001
		MC1R	-0.3873	0.001
		GNB2	-0.3754	0.001
		CHMP7	0.3863	0.001
		TUBA4A	-0.3975	0.001
		MC1R	-0.3873	0.001
		GNB2	-0.3754	0.001
		FGR	0.3613	0.002
		NFKB1	0.3627	0.002
		SV2B	0.364	0.002
		NT5E	-0.3511	0.002
		PCM1	0.3535	0.002

		DYNC1I1	-0.3671	0.002
		AKT1	-0.3539	0.002
		CR1L	0.3435	0.003
		CIITA	0.3478	0.003
		BTNL2	0.3507	0.003
		IMPDH1	-0.3478	0.003
		BTN2A2	0.339	0.004
		PTGES2	-0.3351	0.004
		ATP6V1D	-0.3315	0.004
		IRS1	-0.3323	0.004
		TUBA3E	0.3314	0.004
		TUBB2C	-0.3324	0.004
		ITK	0.3256	0.005
		RAP1GAP2	-0.3251	0.005
		EIF4A3	-0.3293	0.005
		DIAPH1	-0.3279	0.005
		AP1M2	-0.3278	0.005
		PSMD7	-0.3308	0.005
		FBXW10	0.3201	0.006
		ADAM8	0.3239	0.006
		CD40LG	0.3188	0.006
		HCK	0.3223	0.006

NT5E	-0.3511	0.002
DUSP16	0.3425	0.003
TDRD10	0.3419	0.003
TAF4B	0.3427	0.003
IMPDH1	-0.3478	0.003
CCNK	-0.3472	0.003
CHMP5	-0.3419	0.003
C14orf93	0.3502	0.003
CCNK	-0.3472	0.003
CHMP5	-0.3419	0.003
UVRAG	0.3331	0.004
PDZD3	0.3388	0.004
IRS1	-0.3323	0.004
TUBA3E	0.3314	0.004
TUBB2C	-0.3324	0.004
SIGMAR1	-0.3289	0.005
TCEB3	-0.3282	0.005
AP1M2	-0.3278	0.005
HGS	-0.3251	0.005
PSMD7	-0.3308	0.005
HGS	-0.3251	0.005
CD40LG	0.3188	0.006
DBP	0.3211	0.006

ANKRD6	0.3175	0.007
PLAC8	0.3128	0.007
TRIM9	-0.314	0.007
UBE2R2	-0.313	0.007
TUBB8	-0.3169	0.007
MET	-0.3148	0.007
TUBB3	-0.3151	0.007
CD74	0.3096	0.008
TNFRSF17	0.3091	0.008
APC2	-0.3118	0.008
C9	0.3066	0.009
EIF4A1	-0.3058	0.009
ATP6V1H	-0.3054	0.009
PTPN6	0.303	0.010
PXN	-0.3009	0.010
IL31RA	-0.3004	0.010
AP1S1	-0.3028	0.010

		HCK	0.3223	0.006								
		SNAP25	0.3184	0.006								
		PRKAR2B	0.3217	0.006								
		CYSLTR1	0.3137	0.007								
		IL11RA	0.3154	0.007								
		CORO1A	0.3155	0.007								
		FSHB	0.3155	0.007								
		VHL	0.3162	0.007								
		MET	-0.3148	0.007								
		TUBB3	-0.3151	0.007								
		CD3D	0.3091	0.008								
		APOBEC3G	0.3077	0.009								
		NOX1	0.3049	0.009								
		ATP6V1H	-0.3054	0.009								
		GNG2	0.3029	0.010								
		CPSF4	-0.3037	0.010								
		AP1S1	-0.3028	0.010								
		CDK9	-0.3027	0.010								

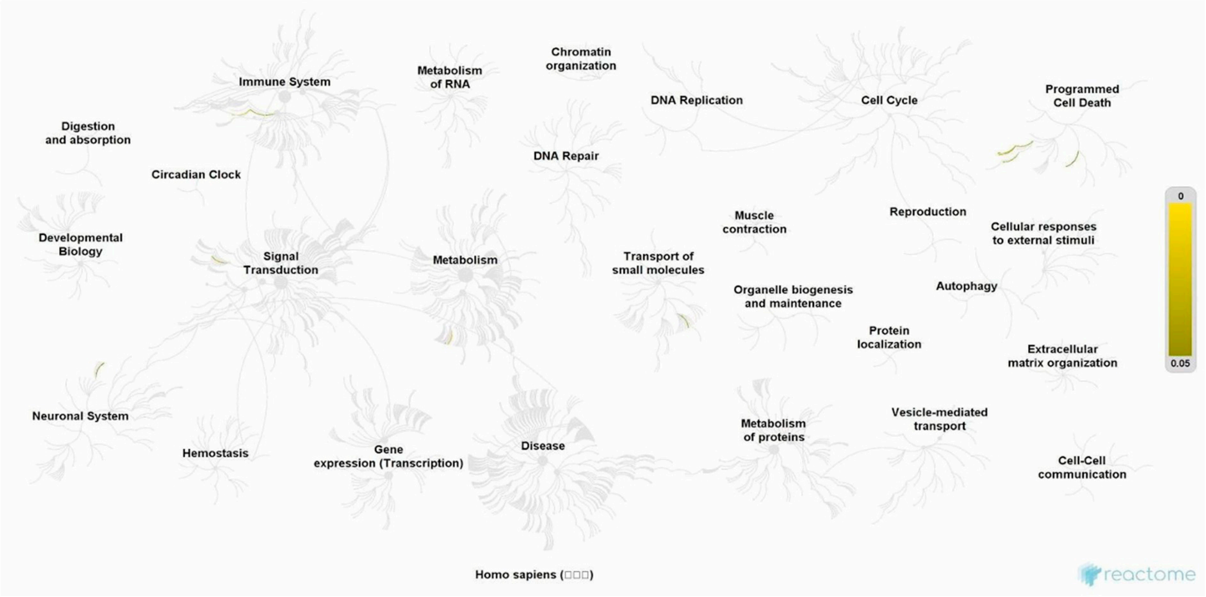

Suppl. Figure S2. The Reactome graphical map illustrates the known biological processes and pathways involving EGOT among HPV-negative patients. Significant associations are highlighted with yellow lines; Spearman correlation with a $p < 0.05$.

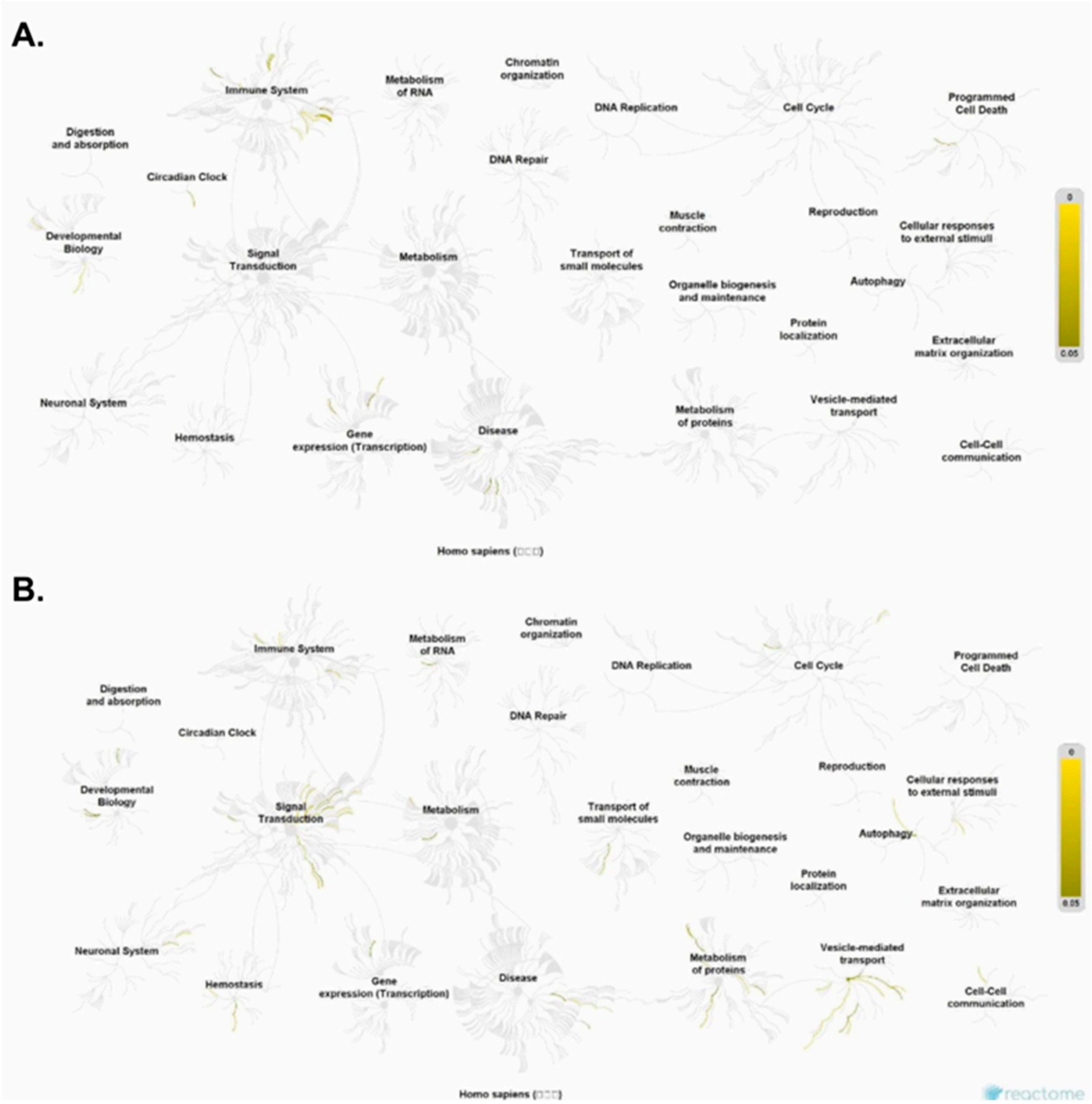

Suppl. Figure S3. The Reactome graphical map illustrates the known biological processes and pathways involving EGOT among HPV-positive patients. Significant associations are highlighted with yellow lines; Spearman correlations (positive, A; negative, B) with a $p < 0.05$.
